# Optimal Design of a Leaf Flexure Compliant Mechanism Based on 2-DOF Tuned Mass Damping Stage Analysis

**DOI:** 10.3390/mi13060817

**Published:** 2022-05-24

**Authors:** Yung-Sheng Chang, Vu N. D. Kieu, Shyh-Chour Huang

**Affiliations:** 1 Department of Mechanical Engineering, National Kaohsiung University of Science and Technology, Kaohsiung 80778, Taiwan; f107142150@nkust.edu.tw; 2 Faculty of Engineering and Technology, Nguyen Tat Thanh University, 300a Nguyen Tat Thanh, Ward 13, District 4, Ho Chi Minh City 700000, Vietnam

**Keywords:** leaf flexure, tuned mass damper, finite elements method, Taguchi’s optimization method

## Abstract

This study proposed an innovative design of a leaf flexural-based 2-DOF tuned mass damping stage that can be integrated into a micro-electromechanical system precision positioning stage to reduce the displacement response of the precision positioning stage excited by a specific vibration frequency and to achieve the damping effect and vibration reduction without adding viscous damping materials. A prototype that conforms to dual-axis decoupling and has 2-DOF translation capability was designed using parallel and vertical arrangements of a leaf flexure. The Taguchi design method and the finite element method were used on the relevant design parameters of the primary mass stage to determine the best size configuration for the maximum off-axial stiffness ratio and the parameters of the tuned mass damper closest to the natural frequency of the primary mass stage with the minimum deflection. In addition, an optimization module, based on a genetic algorithm (GA), was used to optimize the design of the flexure size of the tuned mass damper. Finally, experiments were conducted, the vibration displacement response of the primary mass stage was observed, and the effect with or without the addition of tuned mass damping on the system vibration response was compared. The results indicate that the tuned mass damper can effectively reduce the response amplitude of the stage, where the maximum reduction rate in the experiment was 63.0442%, and the mass of the damper was highly positively correlated with the amplitude reduction.

## 1. Introduction

The development of science and technology has enabled the emergence of various automated machine tools to reduce pressure on humans and increase production capacity. Among them, micromechanical systems (MEMS) with high-precision requirements have received the most attention from research and design personnel in various fields. Conceptually, the design of MEMS is very different from the traditional mechanism designs. Compared with traditional mechanism designs, different components can be assembled separately to produce different motions; MEMS is used less due to the limitation in size. Therefore, introducing the design concept of compliant mechanisms is important to integrate different motion characteristics into a microelectromechanical system. The compliant stage is one of the key subsystems in precision motion control applications, and the flexure hinge is the key element that affects the motion performance of the entire flexure stage.

Liu et al. (2016) [1] developed a new type of V-shaped flexure hinge (QVFH) using the topology optimization method, which can provide a convex shape at both ends and a short-blade flexure in the middle. Due to its higher rotational accuracy, better ability to maintain the rotational pivot position on the flexure element, and less flexibility compared to the traditional V-shaped flexure hinges, it is suitable for designing small-deformation and high-precision flexure mechanisms. Blade-type deflection is usually used to improve the flexibility of a flexible hinge to achieve a large deformation motion. Dragonfly wings inspired [2] to develop an application to position the photographic lens of a nano-indentation tester. The system is based on a dragonfly-inspired compliant joint (DCJ) that integrates the Taguchi optimization method, the adaptive neuro-fuzzy inference system (ANFIS), and the JAYA algorithm to optimize the design of the size parameters to meet the performance requirements of DCJ maximum displacement, higher natural frequency, and minimum stress value. However, the low damping properties of general metal materials can easily affect the dynamic response of the system and limit its performance.

Moreover, the numerical model was simulated by using the finite element method, incorporating the fuzzy logic system, and an ANFIS for a compliant gripper mechanism was introduced [3]. In particular, [4] proposed a theoretical model of a blade-type flexible hinge with a damping layer and conducted free vibration and modal analysis. In this study, the experiments have confirmed that flexible hinges with constrained damping layers can effectively suppress vibration, and an optimization design of a bridge-type compliant mechanism with flexible hinges was investigated [5,6]. In addition, Dao et al. (2017) [7] used polydimethylsiloxane (PDMS) to fill the curved thin-walled flexible joints (compliant thin-walled joint, CTWJ), which improved the equivalent rigidity and displacement performance of flexible joints and increased the strain energy better than traditional flexible joints. Moreover, the first and most basic performance requirement of a flexible platform is to ensure that each degree of freedom can move independently without interference from the other degrees of freedom, that is, to achieve the decoupling characteristics of the motion axis. A kinematic separated and simple structure was proposed (Xavier et al., 2015) [8], which allows the XY-axis displacement platform to be integrated into the precise positioning system of the hybrid micro-product assembly system. The parallel and vertical arrangements of the blade-type deflection ensure off-axis rigidity, and its dynamics and kinematic characteristics were analyzed. The experimental results show that the coupling degree of the motion axis was less than 2.36%, and the off-axis stiffness ratio was 2.3. Li et al. (2009) proposed a flexible hinge with XY motion axis decoupling and a complete decoupling of the input and output. The XY fully decoupled parallel stage (TDPS) can effectively isolate the axial and lateral stress, as generated by the moving stage on the flexible stage, as well as the reaction force, as caused by the drive using double composite parallelogram flexures [9]. For the purpose of integrating a compact lever-type displacement amplifier to meet the output displacement performance requirements, [10] analyzed the dynamic performance of the displacement amplifier and used the Taguchi optimization method to optimize the geometric parameters of the amplifier structure to achieve the maximum response. The frequency achieved a stable amplification vibration, and the results show that the amplifier structure is stable at an amplitude of 2.07 mm at 207.27 Hz. However, the flexible platform or flexible amplifier made of metal materials also has the problem of low material damping.

In [11,12], the flexible platform was used to predict the strong elastic deformed part of the position. By adding liquid PDMS rubber, as a concept similar to that of the shear damper, and combining it with 3D plastic laminate manufacturing technology to achieve a dynamic performance comparable to that of the metal flexible platforms, [13] created a flexible guiding mechanism, which combined the comb-like structure for shear damping to improve the structural damping ratio; thus, they improved the control bandwidth; then, they integrated ABAQUS, MATLAB, and Python to optimize the size parameters of the comb structure to achieve larger modal damping and a smoother frequency response. In addition, a modified velocity command was used to position the platform through command shaping, which adopted a vibration suppression method to reduce the residual vibration in the base structure to elevate the positioning platform accuracy [14].

The concept of tuned mass damping was first used by Frahm in 1909 to control the vibration of a ship’s hull and reduce the rolling motion of the ship [15]. As a passive damping system, it is mainly designed to absorb the vibration energy of a building, which is added by external vibration sources, and thus reduce the shaking of the building. The magnitude of time reduces the discomfort of the people inside the building. The advantages of tuned mass damping are that it can elastically adjust the operating frequency range according to the main structure; it has a design flexibility that makes it possible to install it without affecting the design of the main structure; and it is compact, reliable, efficient, and has low maintenance costs. Therefore, it is widely used by engineering designers to absorb the vibration of external excitation. In 2013, Gordon and Matthew [16] developed a set of optimal passively tuned mass damping systems by creating a finite degree of freedom model for four offshore floating wind power platforms. In addition, Zhuang et al. (2000) [17] used semi-active tuned mass dampers to reduce vibrations during choir performances in church balconies. The vibration caused by the parameters was analyzed to determine the optimal hardware configuration and simulate the nonlinear control equation of the motion to evaluate the damper performance. In addition, the semi-active tuned mass damper, as based on Lyapunov control, was used to reduce the RMS accelerations by 36% and 41%. Regarding RMS displacement, based on the inverse principle of tuned mass, [18] redesigned the cantilever model and used the numerical model to analyze the Σ-shaped spring that constitutes a double resonance mechanism and applied the dynamics of the drive unit. The model designed the entire movement and, finally, proposed a new mechanism for the Braille display unit.

In optimization algorithms, sampling is a background to feature verification [19]. Three algorithms were developed for a sampling of free-form surfaces at a patch scale. These algorithms take each patch on the surface as a separate unit and distribute the points in two steps. In each step of sampling, the maximum difference between the model and the surface which was created using those sampled points was determined. The algorithms converge faster to the value of the maximum dimensional error compared to the two existing sampling strategies. Additionally, the number of points can be controlled, and the optimal number of points is obtained. Moreover, in order to develop the accuracy and efficiency of metamodel, in [20] the scheme of the particle swarm optimization intelligent sampling (PSOIS) was developed. In this study, an intelligent method was able to guarantee that the sampling search was in the right direction. However, the feasible region of the bounds of the design variables would have been constrained. To validate of the accuracy of the developed method, the Rosenbrock function was implemented as a benchmark, and the results show that for highly non-linear problems with multiple parameters, the method is able to produce remarkable metamodels. Compared with the other response surface methodology (RSM), the boundary and best neighbor sampling (BBNS) scheme was introduced [21]. As with the study in [20], this method can guarantee that the sampling search is in the right direction, but in a pre-definite interval of design-variable space. In addition, the Rosenbrock function was successfully approximated by the methodology; correspondingly, the response surface (RS) appropriateness can be well predicted by analysis of variance (ANOVA). In addition, the optimizations using metamodels were introduced. Three popular metamodeling methods, including Kriging (KRG), radial basis function (RBF), and least square support vector regression (LSSVR) were considered [22]. By using multidisciplinary metamodeling techniques, the efficiency of optimization for the high-dimensional problem has been improved. In addition, the comparative study of three methods, the balanced scorecard, the GIMSI, and the SKANDIA’s NAVIGATOR, based on metamodels, was presented [23] in order to choose the best guarantee for a common set of criteria.

In recent years, vibration absorption has been an interesting research topic in structural dynamics. An overview on antiresonance assignments through the active or semi-active tuned mass tampers (TMD) was introduced [24]. This paper compared the most important approaches and the methods exploiting the concept of control theory without adding new degrees of freedom into the system. In [25], a generic tuned mass damper inerter (GTMDI) is presented. In this study, an intrinsic effect of the reduction in the input energy of the structure based on GTMDI is proved: the inerter reduces the input energy, which is transmitted into the controlled structures. Moreover, there are the benefits of the GTMDI with two separating inerters, which optimized the inertances to guarantee the displacement control demand with a minimized energy cost. However, the performance of the TMD designs is quite sensitive to slight deviations in the model parameters. Therefore, a methodology to optimally design a set of spatially distributed Tuned Mass Dampers (TMD) is proposed [26]; the results indicate that the methodology proposed could be used to design spatially distributed TMDs for other engineering structures.

As one of the sub-systems designed based on compliant mechanisms in micro-electromechanical systems, the micro-precision positioning stage is mainly used to connect the driver with the photographic system or the probe system and provide single-degree-of-freedom rotation or two-degrees-of-freedom displacement devices. A precision miniature positioning stage can use series, parallel, or vertical arrangements to combine compliant components and rigid body components, in order that the characteristics of the compliant mechanism can effectively reduce the tolerance problems caused by the components or the backlash and wear caused by drive control errors, which can increase the service life of the MEMS and improve the control bandwidth and performance accuracy. In addition, control of the vibration response is particularly important in the design of the micro-precision positioning stages. Different vibration excitation frequencies not only cause different modal responses of the compliant mechanism, which affects the accuracy and performance of the system, but they can also easily reduce the fatigue life of the compliant mechanism or even lead to system failure. Therefore, the most common method to address this issue is to increase the damping ratio of the miniature precision positioning stage to absorb the excitation vibration energy.

Based on the design concept and vibration reduction principle of the compliant mechanism, this paper proposes an innovative blade-type deflection two-degree-of-freedom tuned mass damping that can be integrated or added to the precision positioning stage of the micro-electromechanical system. The purpose is to reduce the displacement response of the precision positioning stage excited by a specific vibration frequency and achieve the damping effect and vibration reduction without adding viscous damping materials. The conceptual design of the stage optimizes the deflection size and the natural flatness of the tuned mass damper and designs a finite element analysis environment and vibration experiments to verify the feasibility of the damping stage and its vibration suppression effect. The main contributions of this paper can be summarized as follows:

1. A design of a leaf flexural-based 2-DOF tuned mass damping stage that can be integrated or attached in a micro-electromechanical system precision positioning stage is proposed. The purpose is to reduce the displacement response of the precision positioning stage excited by a specific vibration frequency and achieve the damping effect and vibration reduction without adding viscous damping materials.
2. The Taguchi optimization design method and the modal analysis of the FEM are used, and the L9 orthogonal table experiment is performed on the relevant design parameters of the primary mass stage to find the best size configuration for the maximum off-axial stiffness ratio.
3. The optimization module based on the genetic algorithm (GA) was used to find the best design parameters of the tuned mass damper closest to the natural frequency of the primary mass stage at the minimum deflection.
4. The experiment was conducted to verify the performance of the proposed methods and to compare the effect with or without the addition of tuned mass damping on the system vibration response.

The structure of the article is organized as follows. The prototype design of the leaf flexure-based 2-DOF tuned mass damping stage and the optimization problems are introduced in the second section. Next, the simulations were implemented based on the finite element method, and experiments were carried out to evaluate the impact of the vibration. Then, the simulation and experiment results are discussed. Finally, the main contributions of the article are presented in the conclusion section.

## 2. Modeling and Optimization Problem

### 2.1. Prototype Design of the Leaf Flexure-Based 2-DOF Tuned Mass Damping Stage

**Figure 1:**
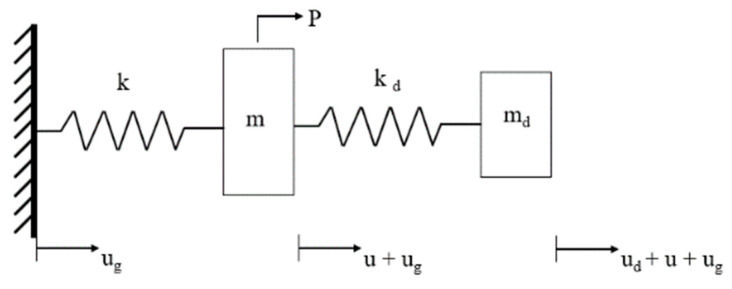
Undamped Tuned Mass Damping System.

Consider the SDOF undamped system, as shown in Figure 1, with m and md as the mass of the primary structure and the tuned mass damper, respectively; k and k_d_ represent the rigidity of the main structure and the tuned mass damping, respectively; u, u_g_, and u_d_ represent, respectively, the ground displacement, the relative displacement of the primary structure, and the tuned mass damper, and p represents the excitation signal [15].

The equations of motion for the primary structure and tuned mass damper can be established separately

(1) $$\{\begin{array}{c} m(\ddot{u}{+a}_{g})+\mathrm{ku}-k_{d}u_{d}=p\\ m_{d}\left({\ddot{u}}_{d}+\ddot{u}{+a}_{g}\right){+k}_{d}u_{d}=0 \end{array}$$

Given that the external excitation source p is a sine wave function $p=\bar{p}\sin\Omega t$ with a cyclic frequency Ω, where $a_{g}$ is the absolute acceleration of the system to the ground, which can be expressed as $a_{g}={\bar{a}}_{g}\sin\Omega t$, then, the displacement response of the primary structure and the tuned mass damper can be expressed as

(2) $$\{\begin{array}{c} u=\bar{u}\sin\Omega t\\ u_{d}={\bar{u}}_{d}\sin\Omega t \end{array}$$

Substituting Equation (1) into Equation (2), we obtain

(3) $$\{\begin{array}{c} (k-m\Omega^{2})\bar{u}-k_{d}{\bar{u}}_{d}=\bar{p}-m{\bar{a}}_{g}\\ -m_{d}\Omega^{2}\bar{u}+(k_{d}-m_{d}\Omega^{2}){\bar{u}}_{d}=-m_{d}{\bar{a}}_{g} \end{array}$$

It can be obtained after simultaneously solving

(4) $$\{\begin{array}{c} \bar{u}=\frac{\bar{p}}{k}[\frac{1-{\left(\frac{\Omega }{\omega_{d}}\right)}^{2}}{B}]-\frac{m{\bar{a}}_{g}}{k}[\frac{1+\frac{m_{d}}{m}-{\left(\frac{\Omega }{\omega_{d}}\right)}^{2}}{B}]\\ {\bar{u}}_{d}=\frac{\bar{p}}{k_{d}}(\frac{\frac{m_{d}}{m}{\bar{a}}_{g}}{B})-\frac{m{\bar{a}}_{g}}{k_{d}}(\frac{\frac{m_{d}}{m}}{B}) \end{array}$$

Among them, $B=[1-{\left(\frac{\Omega }{\omega }\right)}^{2}][1-{\left(\frac{\Omega }{\omega_{d}}\right)}^{2}]-\bar{m}{\left(\frac{\Omega }{\omega }\right)}^{2}$; now, considering that the goal is to minimize the response $\bar{u}$ under the condition of isolating the primary structure from the ground acceleration, set $1+\frac{m_{d}}{m}-{\left(\frac{\Omega }{\omega_{d}}\right)}^{2}=0$, and it can be found after simplifying that

(5) $$\omega_{d}=\frac{\Omega }{\sqrt{1+\frac{m_{d}}{m}}}$$

Because $\frac{m_{d}}{m}$ must not be a negative value, as long as the mass ratio of the tuned mass damper to the primary structure is smaller, the natural circulation frequency of the tuned mass damper is closer to the natural circulation frequency of the excitation source, and finally, the stiffness $k_{d}$ of the tuned mass damper can be derived as

(6) $$k_{d}={\omega_{d}}^{2}m_{d}=\frac{\Omega^{2}{\mathrm{mm}}_{d}}{{m+m}_{d}}$$

Therefore, it is certain that the tuned mass damper can only be triggered by a specific excitation frequency, and in general practice, the mass ratio of the tuned mass damper to the primary structure is set from 1% to 10%.

**Figure 2:**
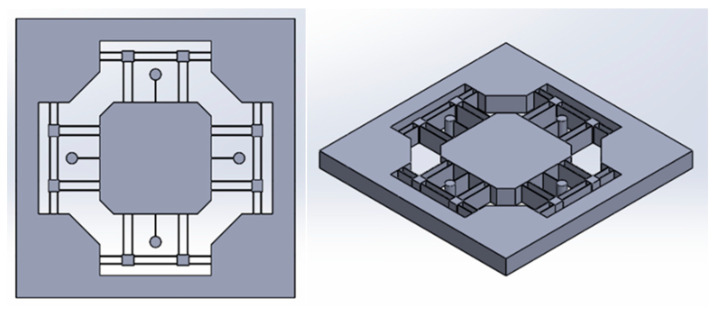
Top view (**top**) and isometric view (**bottom**) of the leaf flexure-based 2-DOF tuned mass damping stage.

Based on the function, it can be divided into four parts: fixed frame, primary mass stage, leaf flexure, and tuned mass damper; the 3D model of the leaf flexure-based 2-DOF tuned mass damping stage is shown in Figure 2. First, the design was made for a single degree of freedom. The concept of fixed-guided beams can constrain the central primary mass stage to produce major linear displacements in a single axis but can rotate relatively on two orthogonal axes. To increase the stiffness of the Z-axis rotation and consider its supportability, a set of parallel leaf flexures was added and moved to the two ends of the primary mass stage. Accordingly, the stiffness of the Y-axis rotation could also be improved. Taking into account the supportability of the Z-axis, an additional piece was connected in parallel at a distance of 8 mm between the four-leaf flexures. To not damage the compliance of the Y-axis translation under the current constraint conditions, a set of parallel leaf flexures, arranged perpendicular to the existing leaf flexure, was added, and the intersection was fixed with rigid blocks. Finally, to allow the main freedom of the primary mass stage to have symmetrical stiffness and compliance, the same leaf flexure configuration was also used in the X-axis and the primary mass stage. The 3D model of the leaf flexure-based 2-DOF tuned mass damping stage is shown in Figure 2. The thickness of the entire platform is 30 mm, and based on the function, it can be divided into four parts: fixed frame, primary mass stage, leaf flexure, and tuned mass damper, where the outermost fixed frame is 250 mm × 250 mm, and trapezoidal blocks extend inward from the four corners to connect and support the leaf flexure structure. The primary mass stage in the middle is 100 mm × 100 mm, and the four ends have a 45-degree lead angle. The fixed frame and the primary mass stage are connected by completely parallel or orthogonally arranged leaf flexures, and the flexural intersections are designed with rigid blocks to provide joint stiffness. In addition, the design of the tuned mass damping was found to correlate highly with the properties of the primary mass stage; thus, before the completion of the optimal design of the primary mass stage to obtain its quality and natural frequency data, this section was only designed for the basic appearance. While the compliant element connected with the tuned mass damper adopted the same abovementioned leaf flexure, it was changed to a single-piece design. In addition, the shape of the part was intended to keep the mass closer to the center of mass. Hence, this study selected a cylinder as the prototype of the tuned mass damper design, as shown in Figure 2, in order to simplify and facilitate the optimal design.

### 2.2. Analysis of Optimal Design

This part used the stage size parameters that make the ratio of the rotation stiffness to the displacement stiffness large in order to achieve the easy translation of the Taguchi design of the primary mass stage. The flexure width, that is, the thickness of the stage, was fixed under the condition of 30 mm, where length L and thickness *t*, which affect the flexural stiffness of the leaf flexure, were divided into three control factors: the primary flexure connected to the primary mass stage and the secondary flexure connected to the fixed frame. The three-level values were set to establish the Taguchi L9 (3^3^) orthogonal array experimental configuration, as shown in Table 1, and SolidWorks was used to model the primary mass stage with nine experimental size configurations to be imported into Ansys for simulation. The Taguchi optimization method, which was developed by Dr. Genichi Taguchi, is an experimental method that can effectively strengthen the quality and solve problems, and its experimental accuracy, application range, and economic effect have always been highly praised. The Taguchi optimization method, which uses tables (orthogonal arrays) to arrange experimental configurations and provide the complete properties of the factors affecting product performance parameters, is different from the general direct trial-and-error method or the full factor method.

**Figure 3:**
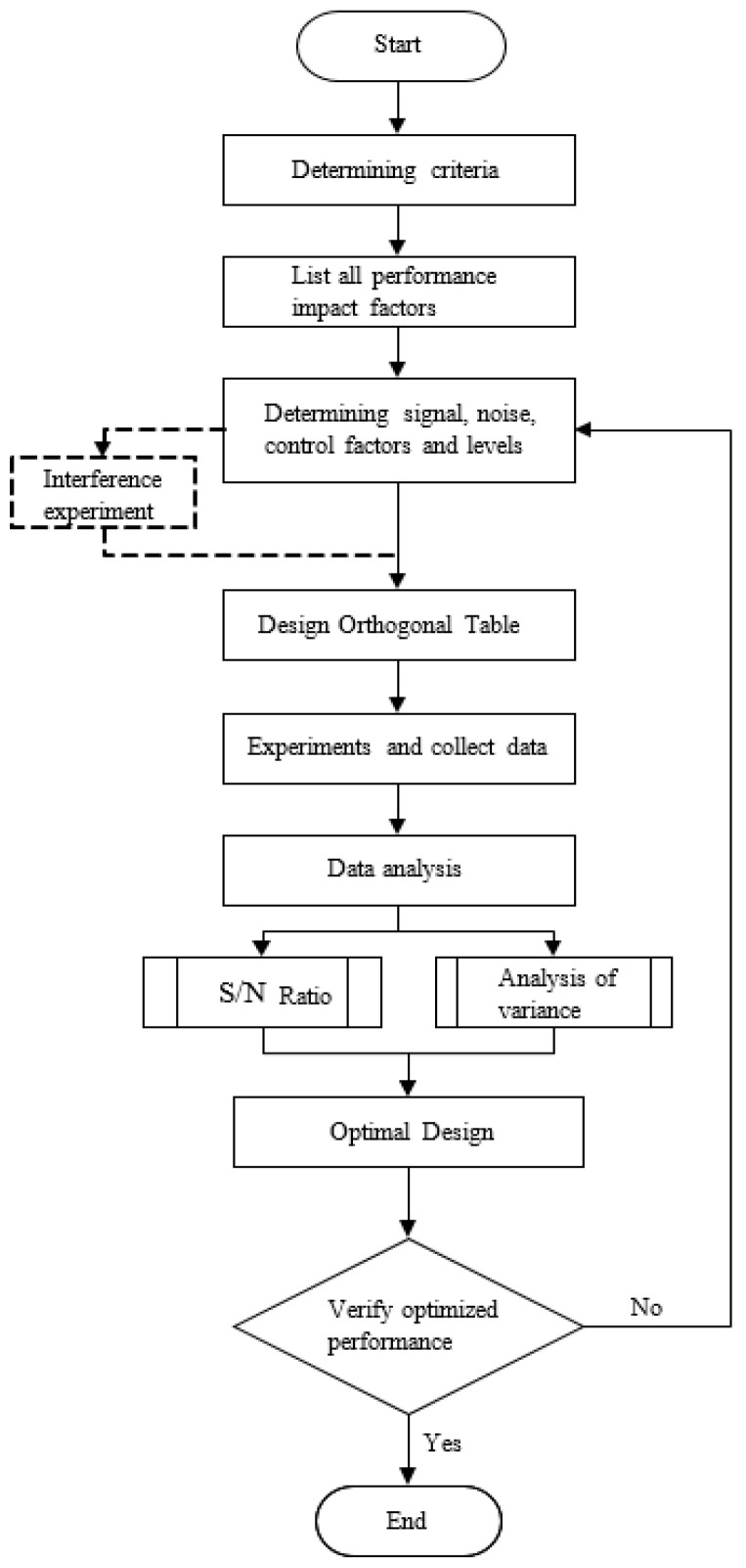
The complete flow chart of the Taguchi optimization method.

**Figure 4:**
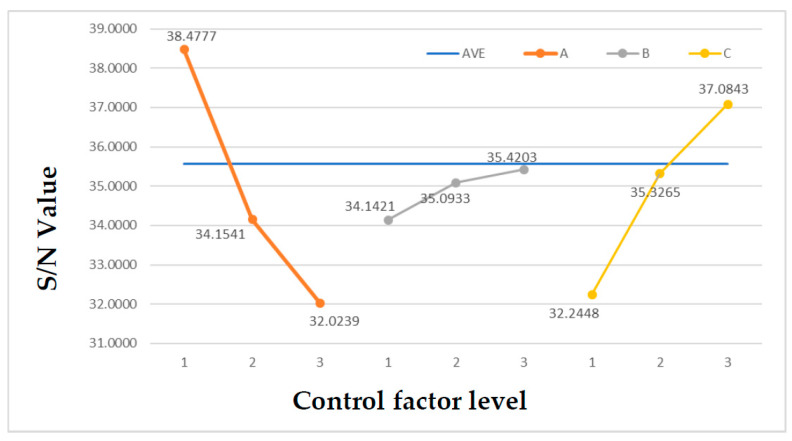
Signal-to-noise (S/N) response graph of the primary mass stage.

The biggest feature of the Taguchi optimization method is the performance of statistical analysis with the least experimental data, where the goal is to determine the optimal configuration of control factors, meaning that the configuration of the factors that maximize the signal-to-noise ratio and the feasibility and efficiency of such experiments are far greater than that of the full factorial method. The operation of the Taguchi optimization method [27] starts by determining the ideal performance criterion of the target and lists all the factors that affect this performance, including signal factors, noise factors, and control factors. Then, several control factors can be adjusted and set for each change level in order to establish the corresponding orthogonal table according to the factors and levels, which is used to systematically configure experiments to record the performance output data. Finally, the S/N ratio and the relationship between the factors can be determined to obtain the optimal control factor configuration result. Moreover, experimental errors can be additionally evaluated by the variance to establish the importance of the control factors on the performance. The complete Taguchi optimization method flow chart is shown in Figure 3. As stiffness can be simply defined as the ratio of force to displacement, the stiffness and force are proportional to the constant displacement; therefore, in order to simplify the operation and accelerate the simulation process, this study used Ansys static structural analysis. The material used for the stage was polylactic acid (PLA), and the mechanical properties are shown in Table 2. Each simulation was given a fixed rotational arc displacement and a linear displacement value; the moment reaction and force reaction were recorded, and their ratio was calculated as the target value for Taguchi optimization. In addition, each test was repeated three times to obtain the experimental error value as the basis for the analysis of variance. The element size was set to 11 mm, according to the results of convergence analysis, and the span angle center was set to “Fine” to render the grid of the geometric edge corners more refined. Regarding the boundary conditions, the periphery of the outer frame was set as a “Fixed Support”, a cylindrical coordinate system with X as the main axis and Z as the rotation axis was defined, and the Y-axis rotation arc displacement was 10 mm to solve the response moment. In addition, a Cartesian coordinate system was used to give a linear displacement of 1 mm on the X-axis to solve the response force. This study used Excel to collect the experimental data, and the relevant statistical formula of the Taguchi Method was used to calculate the variance and signal-to-noise (S/N) ratio of each experiment, as well as the S/N ratio of each factor and level, as shown in Table 3 and Table 4. Finally, the S/N response graph was drawn, as shown in Figure 4. According to the S/N response graph, the control factor to achieve the maximum stiffness ratio was configured as A1B3C3, and the dimensional parameters were a flexure thickness of 1 mm, a primary flexure length of 30 mm, and a secondary flexure length of 22 mm, and the prediction ratio of the torque to the force under the optimal parameter configuration was 102.63. Finally, in order to confirm the reliability of the experiment and the importance of each control factor, the F distribution function built into Excel was used to calculate the confidence level of the error integration.

The natural frequency of the tuned mass damper must be adjusted to be equivalent to the natural frequency of the primary mass stage; therefore, the optimized primary mass stage from the previous section had to undergo modal analysis to obtain its natural frequency. The results show that the first mode and the second mode frequencies that produced translational vibration were 88.15 Hz and 88.255 Hz, respectively. Therefore, the average value of 88.2025 Hz was taken as the target for designing a tuned mass damper. The design size parameters were aimed at the length and thickness that affect the stiffness of the leaf flexure and the radius of the damping cylinder that affects the sensitivity of the tuned mass damper. According to the space constraints of the primary mass stage and the mass ratio of the tuned mass damper to the primary mass stage, the range of the setting size parameters is shown in Table 5. As the Taguchi method can only optimize the design for selected level parameters, it was difficult to effectively approximate the target natural frequency. Therefore, the optimal design of the tuned mass damper used the Ansys Workbench built-in optimization module, which is based on a genetic algorithm, to obtain tuned mass damper size parameters close to the natural frequency of the primary mass stage.

**Figure 5:**
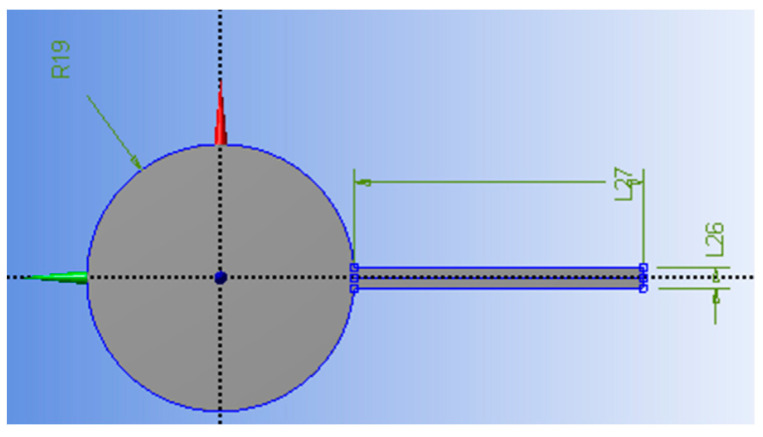
The initial size design model.

First, the Design Modeler was used to design the prototype, and the relevant dimensions were selected as the design parameters, calculation factors, and variance analysis to calculate the contribution of each factor and error; the results are shown in Table 6. The results show that the deflection thickness of factor A had the highest contribution at 59.39%, followed by the deflection thickness of factor C at 34.57%. The overall experimental error of 0.17 contributed only 0.15% to the variation, and the 100% confidence level of all the factors was sufficient to prove its importance and the correctness of this experiment. As shown at L26, L27, and R19 in Figure 5, the initial dimension parameters are given as thickness *t* = 1 mm, length *l* = 14 mm, and cylinder radius *r* = 6.5 mm; then, the modal analysis solver was entered for the initial solution. The pre-processing part also used PLA material in accordance with the simulation of the main mass platform, where the mesh size was set to 11 mm, and the cross-axis center angle was set to “Fine”. Regarding the boundary conditions, the connection area between the tuned mass damper and the primary mass stage was set as a fixed support to finally solve the first modal frequency that produces bending. The initial result of the first modal frequency was 129.81 Hz.

Then, the response surface was used to set the range for the selected design parameters to establish a visualized response surface between each design parameter and the first modal frequency. The response surface module is automatically divided into several groups of design points, according to the parameter range, for the simulation and the solving of the individual first modal frequencies. Table 7 shows the response point results with a tolerance of 0.5. Then, the difference method was used to connect the scattered response points into a continuous surface, which is called the response surface. Finally, the response surface optimization module was used to determine the design point with the target frequency of 88.2025 Hz on the response surface.

**Figure 6:**
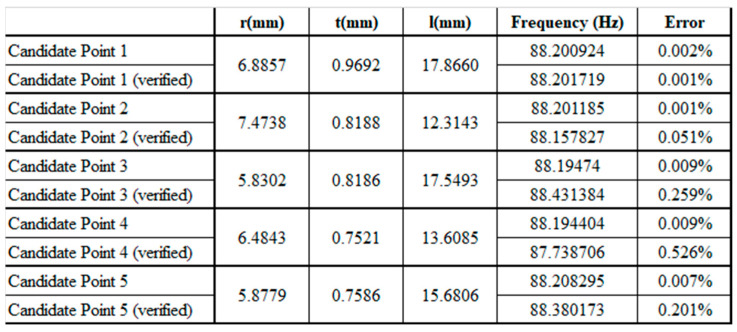
The results of the response candidate points.

Based on the genetic algorithm, the population in each generation is 600 response points for convergence. To avoid repeated searches, after setting 10 candidate response points, 5 candidate points were manually selected with minimum errors, where the target frequency was taken as the reference. The final selected candidate points are shown in Figure 6. The minimum error after verification was 0.001%, and the maximum error was only 0.526%; therefore, candidate point 1 was selected as the optimal design size parameter for a tuned mass damper.

## 3. Simulations and Experiments

### 3.1. Finite Elements Simulation of Dynamic Response

The modal analysis module of the Ansys Workbench was coupled with the harmonic response module to compare the dynamic response distribution of the primary mass stage and the damping stage at different frequencies, and a preliminary evaluation of the vibration reduction characteristics of the leaf flexure tuned mass damping stage was carried out. The boundary conditions of the modal analysis set the outer frame as a fixed support, and the harmonic response was coupled according to the results of the modal analysis. Given a base excitation with a displacement amplitude of 0.1 mm, the frequency sweep range was set from 50 Hz to 100 Hz, according to the modal analysis result, which was set to 5000 divisions to improve the resolution of the response result.

### 3.2. Figures, Tables and Schemes

To verify the optimal design of the leaf flexure-based tuned mass damping stage, this study used a power supply, pulse generator, amplifier, PST, and laser displacement sensor to test its vibration reduction performance. Then, the dynamic response difference between the primary mass stage and the damping stage was compared to judge the feasibility of this conceptual design. First, the aluminum extrusion was used to assemble the linear slides required for the experiment, and the primary mass stage and the damping stage model were modified for the experimental configuration without affecting the function. The M3 screw was opened through a hole at the end position of one side of the platform as the position for connecting the PST. In addition, the M5 screw was opened through holes at the four corners of the platform to fix the position of the V-shaped pulley that matched with the linear slide rail. As an experimental model made with 3D FDM affects the equivalent density and quality of the structure due to the infill density, the central stage and the tuned mass dampers had hollowed out cylindrical holes with a diameter of 10.5 mm and a depth of 20 mm, which were arranged symmetrically to place the counterweight steel balls. Finally, a thin plate structure was established on the central stage in the vertical platform motion direction as the measurement reference position of the laser displacement sensor. In addition, considering the manufacturing size limitations of the 3D printer, the outer frame was made to a depth of 15 mm at the four inward ends.

**Figure 7:**
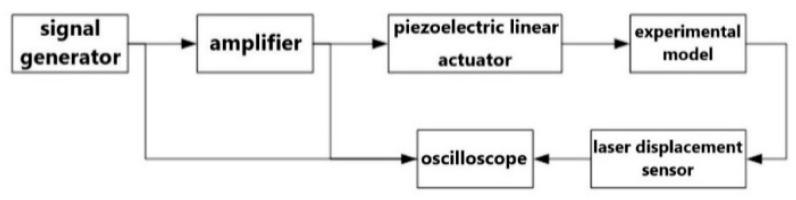
Framework diagram of the vibration experiment.

**Figure 8:**
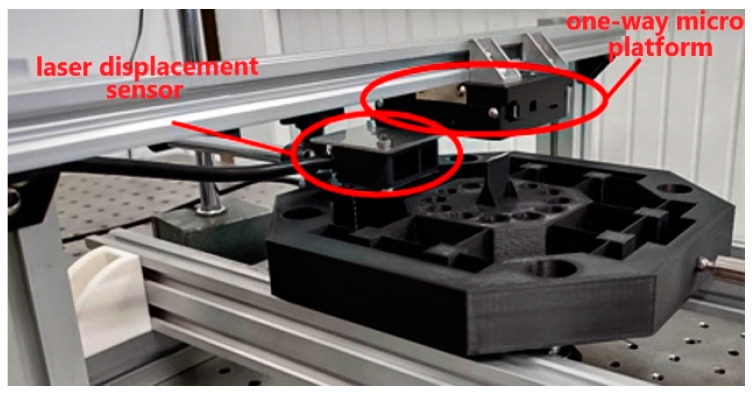
Configuration of the laser displacement sensor.

**Figure 9:**
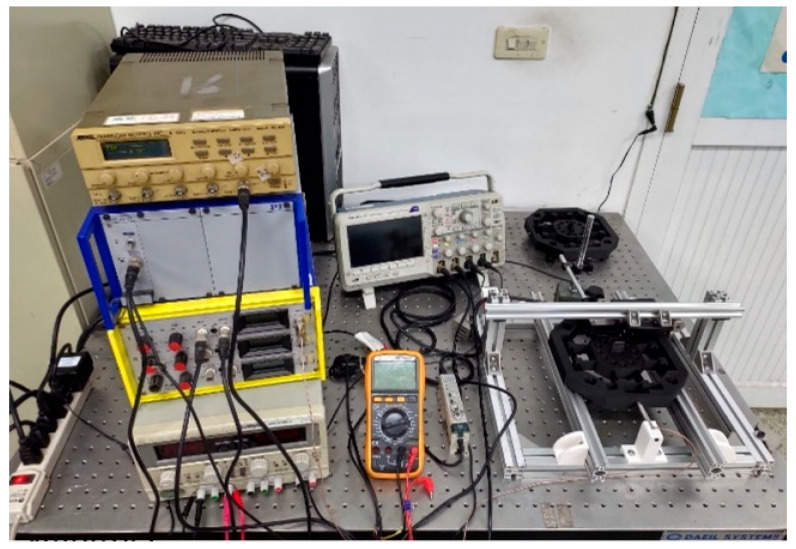
Vibration reduction experiment configuration.

As shown in Figure 7, the pulse generator was first connected to the amplifier, and then, the amplifier was connected to the PST, and finally, the PST was connected to the experimental model with screws to complete the configuration of the input terminal. In addition, each connection terminal was connected in parallel with an oscilloscope to observe the signal of the input model to ensure the stability of each signal transmission process. The output end of the experimental model was mainly measured by the laser displacement sensor, which was first fixed on the unidirectional micro-motion platform, then, it was connected with the U-shaped truss formed by the aluminum extrusion and fixed on the base of the linear slide, as shown in Figure 8, as it was convenient for calibrating the best working position of the laser displacement sensor. Finally, the sensor was connected to the oscilloscope to record the vibration displacement and response frequency generated by the central stage. The complete experimental configuration is shown in Figure 9.

## 4. Results and Discussion

The static structural analysis of the Ansys Workbench was used to verify the motion decoupling of the leaf flexure of the primary mass stage. Regarding the boundary condition, the periphery of the outer frame was also set as a fixed support, and force was applied to one of the X-axis directions of the central platform, which ranged from 100 Newtons to 500 Newtons in increments of 50 Newtons. Then, the displacement solution was set on the same surface to simultaneously record the displacements generated in the X-axis and Y-axis directions to calculate the parasitics, as shown in Table 8. The parasitic was estimated to be only about 0.0032%; thus, the main mass platform has excellent motion decoupling properties to ensure that the main axial motions will not interfere with each other.

**Figure 10:**
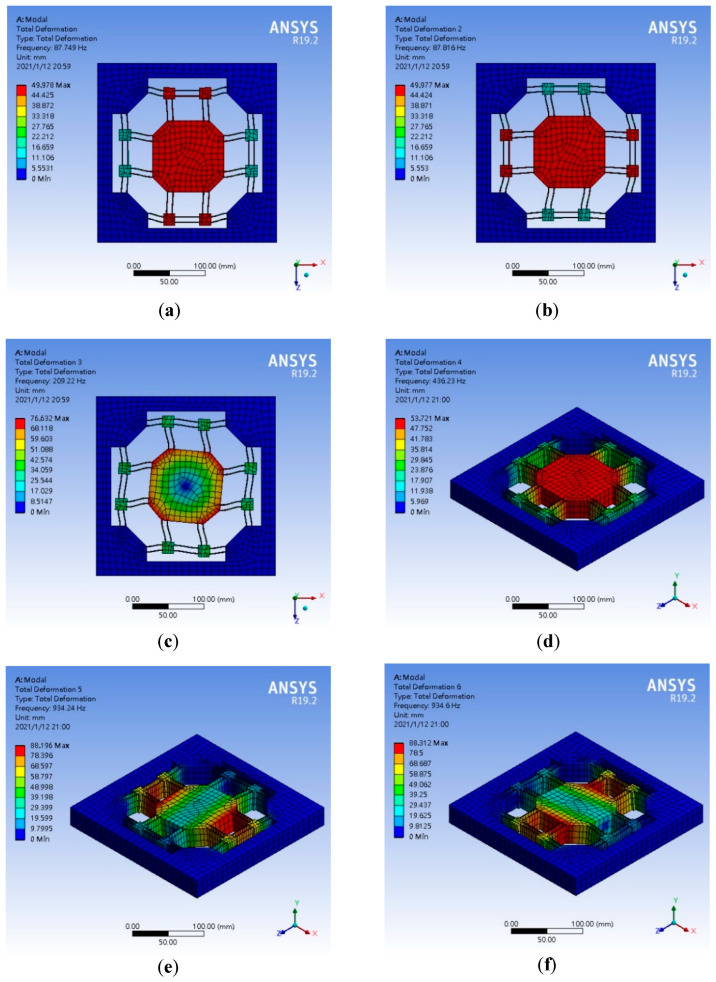
The sixth-order modal analysis results of the primary mass stage. (**a**) Mode 1 (**b**) Mode 2 (**c**) Mode 3 (**d**) Mode 4 (**e**) Mode 5 (**f**) Mode 6.

The sixth-order modal analysis results of the primary mass stage are shown in Figure 10, which shows that the natural frequencies are, respectively, 87.749 Hz (Z-axis translational motion), 87.816 Hz (X-axis translational motion), 209.22 Hz (Y-axis rotational motion), 436.23 Hz (Y-axis translational motion), 934.24 Hz (Z-axis rotational motion), and 934.6 Hz (X-axis rotational movement). The natural frequency of the expected translational motion is less than 100 Hz; thus, this model has a sufficient off-axis stiffness ratio.

**Figure 11:**
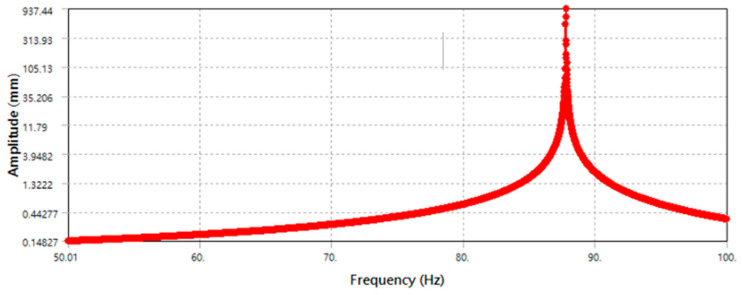
Dynamic response distribution of the primary mass stage.

According to the vibration response distribution diagram, as shown in Figure 11, the main mass platform reached the maximum amplitude of 957.41 mm when the frequency was 87.82 Hz.

**Figure 12:**
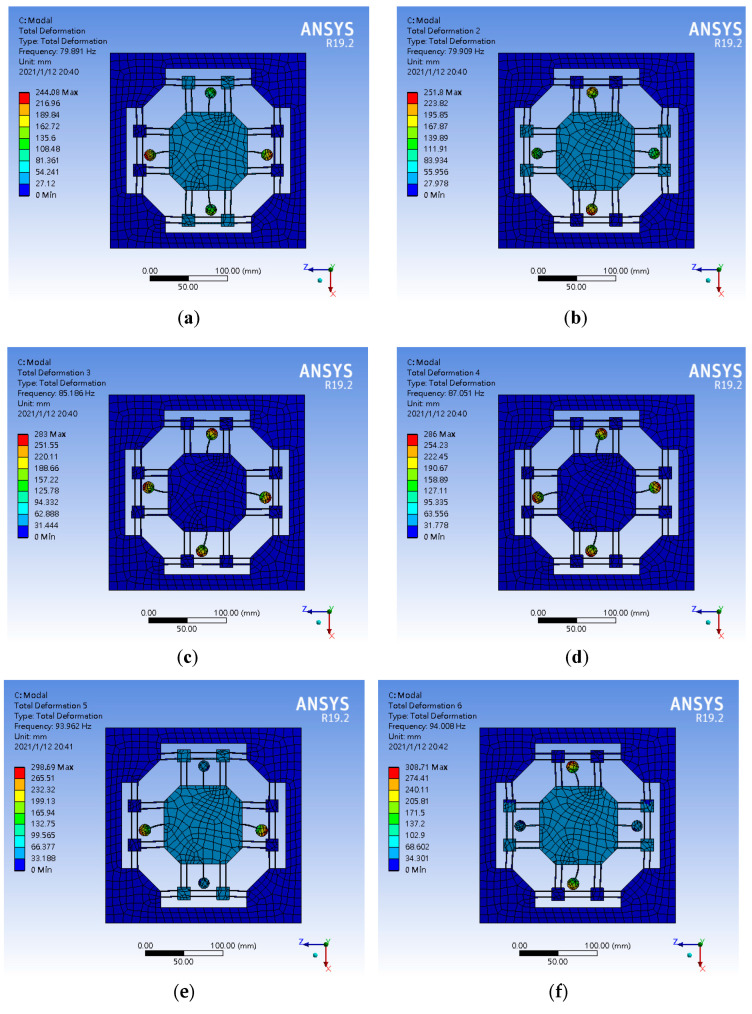
The sixth-order modal analysis results of the tuned mass damping stage. (**a**) Mode 1 (**b**) Mode 2 (**c**) Mode 3 (**d**) Mode 4 (**e**) Mode 5 (**f**) Mode 6.

**Figure 13:**
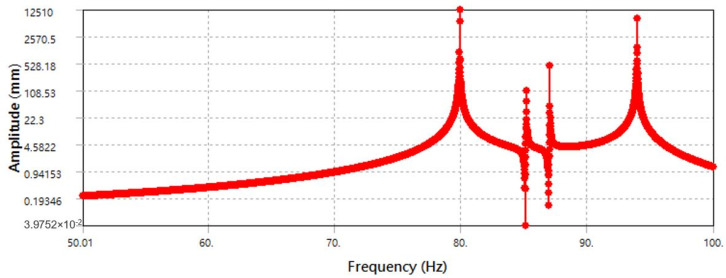
Dynamic response distribution of leaf flexure-based tuned mass damping stage.

According to the results of the sixth-order modal analysis (Figure 12) of the tuned mass damping platform, the natural frequencies are 79.891 Hz, 79.909 Hz, 85.186 Hz, 87.051 Hz, 93.962 Hz, and 94.008 Hz, respectively. Although all the motions are on the XZ plane, mode 1 and mode 2 are the isotropic translational motions of the tuned mass dampers and the primary mass stage, while mode 3 and mode 4 are the independent deflection motions of the tuned mass damper. It can be concluded that the first four modes are mainly caused by the tuned mass dampers, and the main reason for this phenomenon is the difference in stiffness between the tuned mass damper and the primary mass stage. When the stiffness of the tuned mass damper is less than the stiffness of the primary mass stage, the tuned mass damper is first excited by the excitation source to produce a response, which then affects the primary mass stage and runs counter to the purpose of vibration reduction. The motions of mode 5 and mode 6 conform to the form of the tuned mass dampers, and the response amplitude of the primary mass stage to the excitation source is offset by the reverse translation of the tuned mass dampers and the primary mass stage. In addition, according to the vibration response distribution diagram in Figure 13, at the frequency of 79.89 Hz, the maximum amplitude is 12,510 mm; however, this amplitude is caused by the tuned mass dampers. According to the vibration response of the primary mass stage at 93.962 Hz, the maximum amplitude is 483.14 mm.

**Figure 14:**
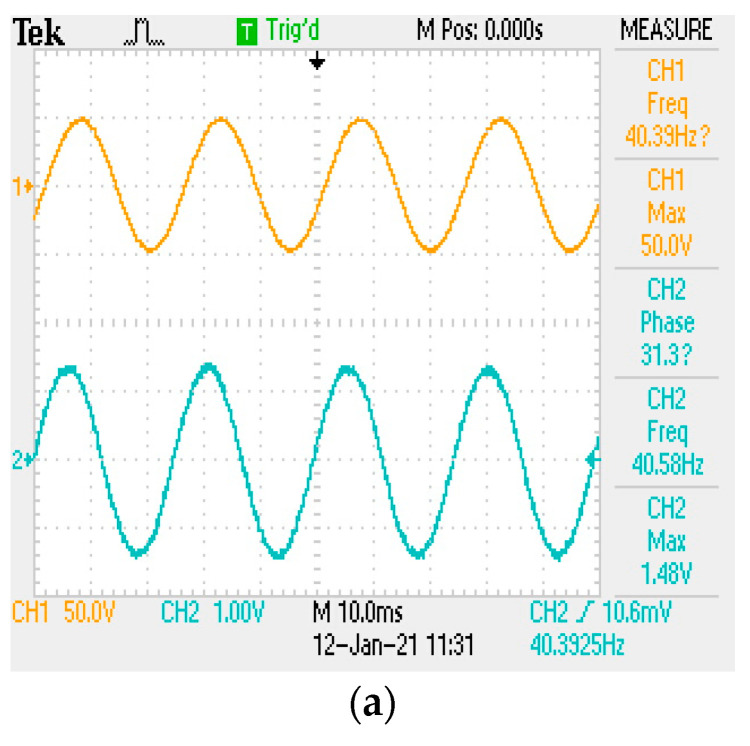

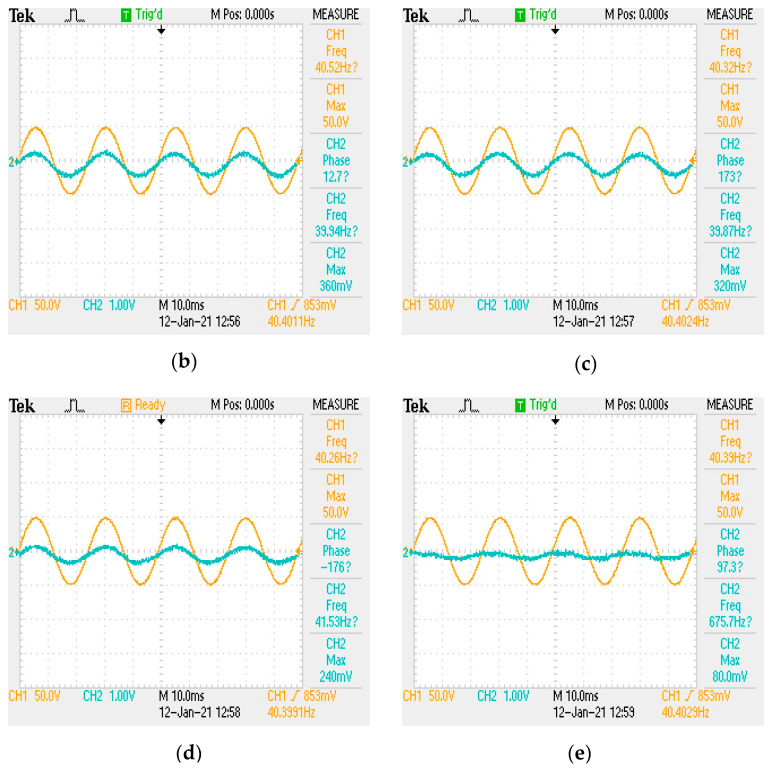
Waveform in the vibration experiment. (**a**) Main mass platform (**b**) Damping platform (8 mm × 1) (**c**) Damping platform (8 mm × 2) (**d**) (8 mm × 2 + 9.5 mm × 1) (**e**) (8 mm × 2 + 9.5 mm × 1 + magnetic iron × 1).

The vibration reduction performance experimental results show that, in the case of sine wave steady-state vibration, when the experimental model reaches the natural frequency at the 40.4 Hz input frequency signal, and resonance occurs, the primary mass stage is without a tuned mass damper. When the maximum input amplitude signal is 50 V, the maximum response amplitude is 1.48 V (Figure 14a). Different mass configurations were carried out for the dampers of the tuned mass damping stage; the 8 mm steel ball was 2.11 g, the 9.5 mm steel ball was 3.52 g, and the magnet was 7.07 g.

The total mass of the damper accounted for 2.398%, 3.878%, 6.346%, and 11.304% of the mass of the primary mass stage, respectively; then, its influence on the vibration of the primary mass stage was observed, as shown in Figure 14b–e. The addition of tuned mass dampers can indeed reduce the response amplitude of the primary mass stage. In this case, as the mass of the damper increased, the maximum response amplitude decreased from 360 mV to 80 mV. Finally, the ratio was used to convert the output voltage amplitude in the oscilloscope into a length representation to create Table 9, which presents the experimental results.

## 5. Conclusions

The results of this paper show that the use of a leaf flexure can increase the deflection angle. The vertical arrangement gave the primary mass stage the characteristics which completely decoupled the translational motion, and the estimated parasitic ratio was 0.0032%. The Taguchi method was used to optimize the design of the leaf flexure geometry of the primary mass platform, and the optimal parameter configuration was derived from the S/N ratio. Moreover, an optimization module was applied, as based on a genetic algorithm, to optimize the design of the flexure size of the tuned mass damper in order for the natural frequency of the tuned mass damper to be equal to the natural frequency of the primary mass stage. After optimization, the dimensions of the tuned mass dampers were the 17.866 mm length, the 0.9692 mm thickness, and the 6.8857 mm radius of the damper cylinder, and the natural frequency was 88.2017 Hz, with an estimated error of only 0.001%. In addition, the finite element simulation results indicated that the modal analysis results of the tuned mass damping stage showed that the first four-order modal motion was mainly caused by the tuned mass dampers. When the stiffness of the tuned mass damper is less than the stiffness of the primary mass stage, the tuned mass damper is first excited by the excitation source to produce a response, which then affects the primary mass stage. Subsequent research on the design of the tuned mass damper may also require that the stiffness of the damper be adjusted to the same stiffness as the primary mass stage, and the experimental results of vibration reduction performance show that the natural frequency of the experiment model was 40.4 Hz, and the maximum output amplitude of the primary mass stage without tuned mass dampers was 91.3043 μm. After adding the tuned mass damper, the output amplitudes were 38.6156 μm, 38.1205 μm, 36.898 μm, and 33.7422 μm under different weight configurations, and the amplitude reduction rates were 57.7067%, 58.2490%, 59.5879%, and 63.0442%, respectively. In addition, the experimental results of the vibration reduction performance confirm that the compliant stage with tuned mass dampers can effectively reduce the response amplitude, and the mass of the damper was highly positively correlated with the amplitude reduction effect.

## Figures and Tables

**Table 1:** Taguchi orthogonal array configuration of the primary mass stage.

Exp	Flexure Thickness (mm)	Main Flexure Length (mm)	Vice Flexure Length (mm)
1	1	20	16
2	1.25	25	19
3	1.5	30	22
4	1	25	22
5	1.25	30	16
6	1.5	20	19
7	1	30	19
8	1.25	20	22
9	1.5	25	16

**Table 2:** Mechanical properties of polylactic acid (PLA).

Density	1250 kg/m^3^
Young’s modulus	3450 MPa
Poisson’s ratio	0.39
Shear modulus	1241 MPa
Elastic modulus	5227.3 MPa
Yield strength	54.1 MPa
Tensile strength	59.2 MPa

**Table 3:** The results of the Taguchi statistics.

Exp	Average	Variance	S/N Ratio
1	56.509	0.1685	35.0424
2	54.650	0.1749	34.7518
3	54.365	0.0975	34.7064
4	111.595	0.3720	40.9529
5	40.349	0.0447	32.1167

**Table 4:** Signal-to-noise (S/N) ratio of each factor and level.

	A	B	C
Level 1	38.48	34.14	32.24
Level 2	34.15	35.09	35.33
Level 3	32.02	35.42	37.08
Level 1	38.48	34.14	32.24

**Table 5:** Tuned mass damper design parameters range.

Parameter	Range
Length (*l*)	10 ≤ *l* ≤ 18
Thickness (*t*)	0.5 ≤ *t* ≤ 1.5
Radius (*r*)	5.5 ≤ *r* ≤ 7.5

**Table 6:** Error integration and variance analysis results.

Factor	SS	DOF	Contribution	Var
A	207.2801	2	59.39%	103.64
B	20.5691	2	5.89%	10.28
C	120.6575	2	34.57%	60.33
Error	0.52	18	0.15%	0.02883
Total	349.0257	24	100.00%	14.54

**Table 7:** Design points and response points.

Exp.	Cylinder Radius (mm)	Flexure Thickness (mm)	Flexure Length (mm)	First Mode Frequency (Hz)
Initial value	6.5	1	14	129.8129
1	5.5	1	14	165.713
2	7.5	1	14	104.4848
3	6.5	0.5	14	46.166
4	6.5	1.5	14	240.6743
5	6.5	1	10	182.1594
6	6.5	1	18	99.4569
7	5.687	0.593	10.748	97.2116
8	7.313	0.593	10.748	65.3892
9	5.687	1.407	10.748	348.5446
10	7.313	1.407	10.748	234.05
11	5.687	0.593	17.252	57.8233
12	7.313	0.593	17.252	40.387
13	5.687	1.407	17.252	212.3165
14	7.313	1.407	17.252	148.4157

**Table 8:** X-axis and Y-axis displacement and the details of parasitics.

Force (N)	X-Axis Displacement (mm)	Y-Axis Displacement (mm)	Parasitics (%)
100	0.8132	2.610 × 10^−5^	0.003209543
150	1.2197	3.915 × 10^−5^	0.003209806
200	1.6263	5.220 × 10^−5^	0.003209740
250	2.0326	6.525 × 10^−5^	0.003210174
300	2.4395	7.830 × 10^−5^	0.003209674
350	2.846	9.135 × 10^−5^	0.003209768
400	3.2526	1.044 × 10^−4^	0.003209740
450	3.6592	1.175 × 10^−4^	0.003209718
500	4.0658	1.305 × 10^−4^	0.003209700

**Table 9:** Frequency and amplitude response of the vibration suppression performance experiment.

Main Mass Platform 142.6 g, Tuned Mass Damper 1.31 g, 8 mm Counterweight Steel Shot 2.11 g, 9.5 mm Counterweight Steel Shot 3.52 g, Magnetic Iron 7.07 g				
Exp. Model	Input Amplitude (μm)	Input Freq(Hz)	Max Output Amplitude (μm)	Output Frequency (Hz)
No TMD			91.3043	40.58
TMD (3.42 g)			38.6156	39.94
TMD (5.53 g)	80	80	38.1205	39.87
TMD (9.05 g)			36.898	41.53
TMD (16.12 g)			33.7422	675.7
